# Hyperconnectivity of the ventromedial prefrontal cortex in obsessive-compulsive disorder

**DOI:** 10.1177/2398212818808710

**Published:** 2018-11-30

**Authors:** Annemieke M. Apergis-Schoute, Bastiaan Bijleveld, Claire M. Gillan, Naomi A. Fineberg, Barbara J. Sahakian, Trevor W. Robbins

**Affiliations:** 1 Department of Psychology, University of Cambridge, Cambridge, UK; 2 Department of Psychiatry, University of Cambridge, Cambridge, UK; 3 Behavioural and Clinical Neuroscience Institute, University of Cambridge, Cambridge, UK; 4 Department of Neuroscience, Psychology and Behaviour, University of Leicester, Leicester UK; 5 School of Psychology and Trinity College Institute of Neuroscience and Global Brain Health Institute, Trinity College Dublin, Dublin, Ireland; 6 Hertfordshire Partnership University NHS Foundation Trust, University of Hertfordshire, Welwyn Garden City, UK; 7 Postgraduate Medical School, University of Hertfordshire, Hatfield, UK

**Keywords:** Ventromedial prefrontal cortex, prefrontal cortex, obsessive-compulsive disorder, resting state, functional magnetic resonance imaging, neuroimaging

## Abstract

Neuroimaging research has highlighted maladaptive thalamo-cortico-striatal interactions in obsessive-compulsive disorder as well as a more general deficit in prefrontal functioning linked with compromised executive functioning. More specifically, dysfunction in the ventromedial prefrontal cortex, a central hub in coordinating flexible behaviour, is thought to be central to obsessive-compulsive disorder symptomatology. We sought to determine the intrinsic alterations of the ventromedial prefrontal cortex in obsessive-compulsive disorder employing resting-state functional connectivity magnetic resonance imaging analyses with a ventromedial prefrontal cortex seed region of interest. A total of 38 obsessive-compulsive disorder patients and 33 matched controls were included in our analyses. We found widespread ventromedial prefrontal cortex hyperconnectivity during rest in patients with obsessive-compulsive disorder, displaying increased connectivity with its own surrounding region in addition to hyperconnectivity with several areas along the thalamo-cortico-striatal loop: thalamus, caudate and frontal gyrus. Obsessive-compulsive disorder patients also exhibited increased functional connectivity from the ventromedial prefrontal cortex to temporal and occipital lobes, cerebellum and the motor cortex, reflecting ventromedial prefrontal cortex hyperconnectivity in large-scale brain networks. Furthermore, hyperconnectivity of the ventromedial prefrontal cortex and caudate correlated with obsessive-compulsive disorder symptomatology. Additionally, we used three key thalamo-cortico-striatal regions that were hyperconnected with our ventromedial prefrontal cortex seed as supplementary seed regions, revealing hypoconnectivity along the orbito- and lateral prefrontal cortex-striatal pathway. Taken together, these results confirm a central role of a hyperconnected ventromedial prefrontal cortex in obsessive-compulsive disorder, with a special role for maladaptive crosstalk with the caudate, and indications for hypoconnectivity along the lateral and orbito pathways.

## Introduction

Obsessive-compulsive disorder (OCD) is a debilitating psychiatric disorder characterised by the presence of intrusive obsessions, compulsions and most often both (American Psychiatric Association, 2013). The neurobiology underlying OCD symptomatology implicates maladaptive thalamo-cortico-striatal (TCS) interactions associated with impairments in cognition and emotional functioning (Alexander et al., 1986; Menzies et al., 2008). Neuroimaging studies have highlighted task-related abnormalities within these TCS loops during cognitive tasks (Abramovitch et al., 2011; Roth et al., 2007; Stern et al., 2012), at rest (Di Martino et al., 2008; Harrison et al., 2009), as well as during symptom provocation (Banca et al., 2015; Nakao et al., 2005).

Advances in elucidating the neuronal substrates of OCD also indicate that the pathophysiology of OCD reflects a deficit in prefrontal functioning linked with reduced cognitive flexibility (Chamberlain et al., 2008; Saxena et al., 1998) and cognitive control (reviewed in Dalley et al. (2011)), impaired response inhibition (Aron et al., 2004), compromised decision-making (Clark et al., 2004) and a deficiency of goal-directed action control over habitual behaviour (Gillan et al., 2015).

Within the prefrontal cortex, dysfunction of the ventromedial prefrontal cortex (vmPFC), a central hub in coordinating flexible behaviour, is thought to play a pivotal role in anxiety-related disorders (Myers-Schulz and Koenigs, 2012), including the TCS neuropathology found in OCD (Clark et al., 2004; Stern et al., 2011). The vmPFC is critical to value updating and self-representation as part of the default mode network (DMN) (Gusnard et al., 2001; Roy et al., 2012). Patients with OCD have shown to exhibit reduced deactivation of the vmPFC during error processing (Stern et al., 2011) and an absence of vmPFC safety signalling in OCD patients during differential threat learning and reversal was found to be predictive of a failure to learn that a previously threatening stimulus had become safe (Apergis-Schoute et al., 2017).

Heightened activity of the vmPFC in OCD patients at rest has been well documented using positron emission tomography (PET) (Whiteside et al., 2004), has been linked to symptom severity and normalised following behavioural and pharmacological treatment (Baxter et al., 1992, 1988; Schwartz et al., 1996) and has been shown to be reduced by deep brain stimulation (DBS) of the subthalamic nucleus (STN), with the amount of reduction correlating with reduced OCD symptomatology (Le Jeune et al., 2010). A possible interpretation of the vmPFC hyperactivation found at rest implicates the default mode network and its involvement in self-referential thinking (Buckner et al., 2008; Gusnard et al., 2001).

Considering the importance of the vmPFC in valuation and flexible behaviour combined with the consistency of vmPFC hyperactivity in OCD, and recent findings from our own group showing an absence of vmPFC safety signalling in OCD (Apergis-Schoute et al., 2017), we sought to determine the intrinsic alterations of this region using resting-state functional magnetic resonance imaging (fMRI). Although previous studies have already revealed increased orbito-frontal connectivity in OCD patients compared to controls (Beucke et al., 2014; De Vries et al., 2017; Harrison et al., 2009, 2013; Sakai et al., 2011), none of these studies have used the vmPFC as a seed region. To investigate vmPFC activity and network connectivity during rest, we employed a resting-state functional connectivity (rs-FC) magnetic resonance imaging (MRI) analysis (first described by Biswal et al. (1995)) with a vmPFC seed region of interest (ROI) based on the hyperactivity and safety signalling impairments found in the same group of OCD patients and matched controls, which includes parts of Brodmann areas 32 and 24 as well as the very medial part of area 10 (Apergis-Schoute et al., 2017). Through rs-FC MRI analysis, we were able to examine temporal correlations of low-frequency fluctuations in blood-oxygen-level dependent (BOLD) signal (Fox and Raichle, 2007), which can be a valuable biomarker in the identification of disease states in psychiatry by providing information about intrinsic alterations in brain connectivity (Rosazza and Minati, 2011).

A recent resting-state study in patients with OCD has highlighted deficiencies in the neural circuits supporting top-down control, showing regional hyperactivation in thalamus, dACC, precuneus and cerebellum, and hypoactivation of posterior temporal regions (Zhang et al., 2011). Moreover, altered cortico-striatal functional connectivity, both with distinctive dorsal and ventral pathways, and fronto-parietal networks have been previously observed during rest (Harrison et al., 2009; Stern et al., 2012). Furthermore, alterations in DMN connectivity have been found in OCD, relating to abnormalities in self-referential processing (Beucke et al., 2014), suggesting a central role of the vmPFC in intrinsic network connectivity.

Based on studies showing a hyperactive vmPFC correlated with impaired task performance in OCD (Apergis-Schoute et al., 2017; Stern et al., 2011, 2013), we hypothesised hyperconnectivity of the vmPFC at rest in OCD with key regions in the TCS loops. By using a predefined vmPFC ROI from our threat reversal paradigm in the same patients, this study provides unique insight into intrinsic vmPFC functioning at rest using whole-brain connectivity analysis.

## Methods

### Participants in resting-state study

A total of 38 OCD patients and 33 matched controls were included in our analysis. Resting-state data were collected for 40 OCD patients and 41 controls, of which we excluded 5 due to excessive motion artefacts (+4 mm in any orientation) and 5 due to other artefacts such as severe ghosting and signal cut-off (8 controls and 2 OCD patients). The majority of patients were right handed but we also included 8 left-handed participants (2 controls and 6 OCD). Eligible participants reported no history of head trauma, neurological disease, substance dependence or contraindications for MRI. Participants provided informed written consent prior to participation in our study, which was approved by the Cambridge Central Research Ethics Committee.

All participants were screened with the Mini International Neuropsychiatric Inventory (MINI) to assess the presence of OCD and absence of any other psychiatric conditions in OCD patients, and to confirm that healthy participants did not suffer from any mental disorder. Patients with hoarding were not included. OCD symptom severity was measured using the Yale–Brown Obsessive-Compulsive Scale (Y-BOCS; Goodman et al., 1989), mood status was assessed using the Montgomery–Asberg Depression Scale (MADRS; Montgomery and Asberg, 1979), anxiety levels using the State-Trait Anxiety Inventory (STAI; Spielberger, 1987) and verbal IQ was quantified using the National Adult Reading Test (NART; Nelson and O’Connel, 1978). Patients were included if they suffered from OCD, were free from any additional axis-I disorders, had a minimum score of 12 on the Y-BOCS and a maximum score of 16 on the MADRS.

In total, 13 patients were unmedicated and 25 medicated, of which 14 patients were taking selective serotonin reuptake inhibitors (SSRIs) only, 1 patient was on antipsychotics alone, while the remaining 10 patients were on a combination therapy (4 also using tricyclics and 6 using antipsychotics). A post hoc analysis of differences between unmedicated (N = 13) and medicated (N = 25) OCD patients revealed some network differences, which we report on. Our main widespread vmPFC connectivity findings were however on the OCD group (N = 38) as a whole, which were unrelated to the subtle medication differences that we found. Demographic and clinical characteristics are depicted in Table 1.

**Table 1.** Demographic and clinical characteristics.

Variable	Controls (N = 34)	OCD (N = 38)	χ2 or t	p-Value
*Demographics*				
Gender (female/male)	16/18	17/21	χ2 = 0.039	0.84
Age (years)	38.3 ± 13.3	40.7 ± 13.1	t = −0.78	0.44
Medicated (yes/no)		25/13		
Years of education	15.9	15.2	t = 1.1	0.28
Estimated verbal IQ	113.8 ± 8.1	111.7 ± 7.4	t = 1.15	0.26
*Clinical characteristics*				
MADRS	1.1 ± 1.1	7.7 ± 7.2	t = −5.14	<0.00001
STAI state	29.1 ± 6.3	44.8 ± 12.4	t = −6.5	<0.00001
STAI trait	31.1 ± 7.4	56.2 ± 13.2	t = −9.43	<0.00001
OCI-R	5.2 ± 4.9	29.9 ± 10.9	t = −11.8	<0.00001
Y-BOCS total		22.3 ± 5.8		
Y-BOCS obsessions		10.2 ± 4.5		
Y-BOCS compulsions		12.1 ± 3.1		

MADRS: Montgomery–Asberg Depression Scale; STAI: State-Trait Anxiety Inventory; OCI-R: Obsessive-Compulsive Inventory – Revised; Y-BOCS: Yale–Brown Obsessive-Compulsive Scale.

### Resting state

During the resting-state scan, participants were told to keep their eyes on a fixation cross and to not think about anything in particular for 10 min (resulting in 300 volumes). A high-resolution structural (magnetisation-prepared rapid acquisition gradient-echo (MPRAGE)) scan was taken prior to this scan for anatomical normalisation.

### fMRI data acquisition (imaging methods)

Neuroimaging data were acquired on a 3T Siemens Magnetom Trio scanner at the Wolfson Brain Imaging Centre at Addenbrooke’s Hospital (Cambridge, UK). Anatomical images were acquired using a T1-weighted protocol (256 × 256 matric, 176 1-mm sagittal slices) using an MPRAGE sequence (TR = 2000 ms, TE = 2.98 ms, TI = 900 ms, flip angle = 78°). A total of 32 interleaved transracial sections of gradient echoplanar imaging (EPI) slices depicting BOLD contrast were acquired parallel to the intercommissural plane with the following parameters: TR (sampling time) = 2000 ms, TE (echo time) = 30 ms, slice thickness = 3 mm, FOV (field of view) = 192 mm, flip angle = 78°, 64 × 64 matrix and 3 × 3 × 3 mm^3^ voxels.

### Pre-processing

Imaging data were pre-processed and analysed using SPM12 statistical parametric mapping (http://www.fil.ion.ucl.ac.uk/spm/software/spm8; Wellcome Institute of Cognitive Neurology, London), MATLAB (version r2015b) and FSL (version 5.0.9).

Each participant’s raw data were slice time corrected (temporal shifts between slices), to correct for temporal differences between slices within the same volume, using the middle slice as a reference. All functional images were spatially realigned to the first volume, using a least-squares minimisation and a six-parameter (rigid body) spatial transformation, after which the mean image of those realigned volumes was spatially normalised to the Montreal Neurological Institute (MNI) brain template image. Subsequently, realigned images were coregistered to each individual subject’s anatomical/MPRAGE scan after co-registration to the SPM-T1 template (Friston et al., 1995).

Anatomical scans were segmented and normalised to the International Consortium for Brain Mapping (ICBM) space template for European brains (innate SPM template) by the unified segmentation approach (Ashburner and Friston, 2005). Normalisation parameters were applied to the coregistered functional images. All previous interpolation options were utilised using a fifth-degree spline. Finally, functional images were then spatially smoothed with a Gaussian kernel (full width at half maximum = 8 mm). Routine inspection was performed using SPM function ‘CheckReg’ and FLSview, to confirm successful pre-processing and ensure the quality of all images.

### Functional connectivity analyses

To assess functional connectivity between the vmPFC and the rest of the brain, we performed a seed-based functional connectivity analysis. Our approach (as described in Lee et al. (2014)) has been used in numerous studies investigating resting-state connectivity (Biswal et al., 2010). Our vmPFC ROI in the vmPFC was based on strong differences in safety signals in this region in these same patients compared to healthy controls (Apergis-Schoute et al., 2017). Our seed was defined as a 5-mm radial sphere centred around ‘−2, 26, −2’ (all coordinates described are specified in MNI space). Our primary objective was to both compare vmPFC connectivity within the vmPFC itself and with the rest of the brain. Second, we used exploratory analyses of three key TCS regions (caudate, thalamus and superior frontal gyrus) as supplementary seeds as these regions were confirmed to be hyperconnected with our primary vmPFC seed, using 5 mm radial spheres around the peak connectivity voxel. Individual voxel-wise mean activations of the brain were generated, and we extracted a time series from our defined sphere. A random-effects general linear model (GLM) analysis was conducted on the BOLD signal resulting from the 10-min rest period (300 volumes).

Analysis of functional connectivity was carried out using statistical parametric mapping software (SPM12) (Friston and Stephan, 2007). Functional connectivity maps were estimated for each subject based on the vmPFC seed as our predictor of interest, and the white matter (WM) and cerebrospinal fluid (CSF) signals as tissue-specific predictors of no interest (nuisance covariates). We used a single CSF regressor (a 3-mm sphere around the coordinates (16 −34 16)) and a single WM regressor (a 3-mm sphere around the coordinates (−28 −24 30)) and confirmed these locations were accurately placed within the ventricle and deep cortical WM, respectively, in each subject. Mean WM and CSF series were then used as nuisance regressors. These nuisance regressors are often used to account for fluctuations unlikely to be related to neuronal activity, as they are usually physiological artefacts (e.g. cardiac and respiratory cycles) (Fox et al., 2005; Fox and Raichle, 2007).

Six motion parameters from the pre-processing were included as nuisance regressors in the connectivity-based GLM analysis as well as to account for head movements. There were no differences between healthy controls and OCD patients in head movement on any of the six motion parameters (p > 0.4). An equal number of participants in each group had 2–4 mm head movements (eight participants in each group), and the remaining participants all had head movements of less than 2 mm in all directions. In addition, we manually checked each scan for spikes. Data were filtered using a high-pass filter (0.008 Hz). Individual SPMs were thresholded using a family-wise error (FWE) correction of pFWE < 0.05 for whole-brain connectivity.

Second-level (between-group) GLM analyses were performed by subtracting either the control contrast from the OCD contrast (OCD > Control) or vice versa (Control > OCD) using one-Sample T-test on the connectivity data of the two groups. Between-group contrasts were thresholded at p < 0.001 whole-brain differences (uncorrected; minimum cluster extent, 10 voxels).

The first eigenvariates were extracted at multiple ROIs, to summarise the temporal correlation in BOLD response of the seed to the ROI (Friston et al., 2006). This enabled us to use a quantifiable measure of connectivity and correlate this with recorded questionnaire data. The nomenclature of brain regions belonging to the MNI coordinates found in the analysis was appropriated using Brodmann’s (1990) regions. Subsequently, the BioImage suite was utilised for the conversion of MNI space to Brodmann’s areas (Lacadie et al., 2007).

## Results

Overall, we found widespread hyperconnectivity of the vmPFC in OCD compared to controls (p < 0.001 uncorrected), including key regions of the TCS loop and the wider vmPFC area. These results replicate previous resting-state findings showing altered network connectivity (Harrison et al., 2009; Stern et al., 2012). Conversely, we found no regions showing increased connectivity with vmPFC in controls relative to OCD patients, indicating an imbalance of widespread vmPFC hyperconnectivity in this disorder. Neither vmPFC autoconnectivity (R^2^ = 0.0273, p = 0.3216) nor vmPFC-caudate connectivity (R^2^ = 0.0079. p = 0.558) correlated with impaired safety signalling of the vmPFC (Apergis-Schoute et al., 2017). Instead, vmPFC hyperconnectivity, as detailed below, correlated with symptom severity measures.

### Autoconnectivity of the vmPFC

**Figure 1.**
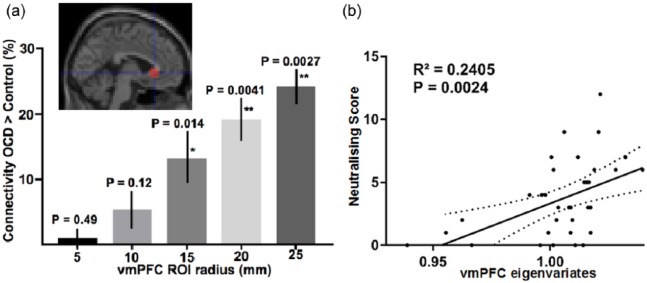
Increased vmPFC autoconnectivity in OCD patients correlates with neutralising scores. (a) Bar plot showing relative increases in autoconnectivity between the vmPFC seed (−2, 26, −2) and the surrounding vmPFC ROI region in OCD (N = 38) in relation to controls (N = 33). OCD patients showed increased connectivity between the vmPFC seed region and the eigenvariates extracted from the surrounding region compared to controls. (b) In OCD patients (N = 38), eigenvariates at the smallest 5-mm vmPFC ROI correlated significantly with neutralising scores (R^2^ = 0.2405, p = 0.0034), remaining significant when correcting for six multiple comparisons (six OCD dimensions) at alpha level = 0.005.

As shown in Figure 1(a), the functional connectivity within the vmPFC is greater in patients with OCD. Using the volume-of-interest function in SPM12, we extracted the first eigenvariates from increasing spheres centred on our vmPFC ROI. We assessed the relative increase in connectivity in OCD by dividing the first eigenvariates of this region for OCD patients by controls’ eigenvariates. We found a relative increase in vmPFC autoconnectivity in OCD that scaled linearly with the size of the radius of the sphere from the seed area (Figure 1(a)). At a 5-mm radius, the vmPFC ROI correlated significantly (r = 0.4904, p = 0.0034) with neutralising scores (Figure 1(b)), while at larger vmPFC ROI sizes starting from a 10-mm radius, a correlation with washing scores (r = 0.4196, p = 0.0108) emerged which persisted across larger vmPFC ROI sizes indicating a link between vmPFC autoconnectivity and washing symptoms, but only the correlation with neutralising scores remained significant when correcting for multiple comparisons.

### Caudate connectivity

**Figure 2.**
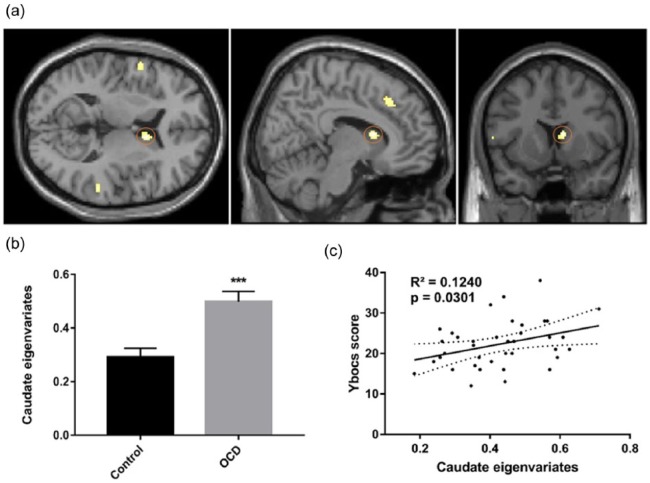
VmPFC-caudate hyperconnectivity in OCD correlates with Y-BOCS scores: (a) axial, sagittal and coronal views (OCD > Control) of vmPFC connectivity with the right caudate (whole-brain differences p = 0.001 uncorrected, cluster threshold = 10 voxels). The Control > OCD contrast showed no significant differences. (b) First, eigenvariates were extracted for these caudate coordinates (10, 12, 10 MNI), which showed a significant (t_69_ = 4.022, p = 0.00012) increase in connectivity in OCD. Error bars denote standard error of the mean. (c) A linear regression analysis depicting a significant (R^2^ = 0.1240, p = 0.0301) correlation between the caudate eigenvariates (20 mm) of OCD patients and their respective Y-BOCS scores.

OCD patients showed increased connectivity from the vmPFC to the caudate (t_69_ = 4.022, p = 0.00012) (Figure 2(a) and (b)). The extracted eigenvariates of the caudate correlated significantly (r = 0.3521, p = 0.0301) with Y-BOCS scores, revealing a specific relationship of excessive functional vmPFC-caudate connectivity with OCD symptomatology (Figure 2(c)).

### Overview of areas hyperconnected with the vmPFC in OCD

Table 2 shows the widespread hyperconnectivity of the vmPFC in OCD, ranking top to bottom based on the maximal z-score of the peak activation. These results are consistent with previous studies implicating brain regions from the TCS loops in OCD, including several regions from the frontal cortex (Chamberlain et al., 2008; Chen et al., 2016).

**Table 2.** Widespread increases in functional connectivity of the vmPFC in OCD.

Connected regions, OCD > Control	BA	Cluster size, voxels	z-score, peak	Primary peak, coordinates
Right caudate	48	59	4.00	10, 12, 10
Fusiform gyrus	37	43	3.98	48, –52, –8
Inferior frontal gyrus	44	36	3.95	−58, 6, 8
Left thalamus	50	67	3.76	−18, –20, –2
Superior frontal gyrus	8	62	3.75	12, 28, 42
Visual association cortex	19	44	3.67	22, –68, –8
Superior temporal gyrus	22	23	3.66	58, –36, 10
Dorsomedial PFC	10	11	3.54	48, 44, 20
Cerebellum	n/a	35	3.50	34, –44, –24
Temporal cortex	21	13	3.48	−52, –48, 6
Primary motor cortex	4	15	3.44	52, –6, 12
Cerebellum	n/a	46	3.43	20, –50, –32
Fusiform	36	10	3.21	28, –32, –14

vmPFC: ventromedial prefrontal cortex; OCD: obsessive-compulsive disorder; BA: Brodmann’s area; PFC: prefrontal cortex.

Brain regions show significant increases in connectivity with the vmPFC seed at (−2, 26, −2). Thresholds were set at p < 0.001 (uncorrected), cluster threshold = 10 voxels.

### Connectivity schematic

Following up from our main hypothesis of increased vmPFC connectivity in OCD, we performed exploratory seed-based connectivity analyses of key regions in the TCS circuitry, for which our results confirmed hyperconnectivity with the vmPFC in OCD patients (thalamus, superior frontal gyrus and caudate; see Table 2) (Harrison et al., 2009).

Table 3 displays the connectivity of the additional three seed regions analysed (right caudate, left thalamus and the superior frontal gyrus). Alongside widespread vmPFC hyperconnectivity, connectivity with the superior frontal gyrus highlights that the OCD brain also displays hypoconnectivity to several prefrontal regions: the dorsomedial prefrontal cortex (dmPFC), dlPFC and the dorsal anterior cingulate cortex (ACC). The connectivity schematic does not include voxels located in white matter bundles that showed a significant correlation with one of our seed regions.

**Table 3.** Functional connectivity of brain regions that showed hyperconnectivity with the vmPFC during resting state.

Seed region	Connected regions, (Per contrast)	BA	Cluster size, voxels	z-score, peak	Primary peak, coordinates
Right caudate		48			10, 12, 10
OCD > CTRL	Amygdala	53/54	15	3.55	−20, –8, –12
CTRL > OCD	Dorsolateral PFC	46	18	3.59	−44, 46, 0
Left thalamus		50			−18, –20, –2
OCD > CTRL	Left caudate	48	102	4.38	−20, 26, 10
	Dorsal ACC^a^	32	250	4.00	20, 40, –2
	Right caudate	48	10	3.85	18, –4, 24
CTRL > OCD	N/A				
Superior frontal gyrus		8			12, 28, 42
OCD > CTRL	Left caudate	48	37	4.00	−6, 18, 10
CTRL > OCD	Agranular retrolimbic	30	161	4.11	0, –46, 14
	Dorsolateral PFC	9	46	4.04	18, 50, 38
	Superior frontal gyrus	8	94	4.01	30, 18, 40
	Angular gyrus	39	120	3.85	−44, –58, 42
	Cerebellum	N/A	170	3.76	26, –68, –34
	Dorsolateral PFC	9	24	3.52	−36, 28, 40
	Dorsolateral PFC	9	37	3.52	32, 36, 38
	Cerebellum	N/A	10	3.48	−26, –74, –42
	Angular gyrus	39	49	3.48	50, –50, 28
	Angular gyrus	39	22	3.47	38, –58, 24
	Globus pallidus	51	35	3.43	−14, 4, 2
	Cerebellum	N/A	44	3.40	−12, –72, –30
	Angular gyrus	39	21	3.40	−42, –66, 22
	Superior frontal gyrus	8	38	3.38	20, 28, 48
	Anterior cingulate	24	13	3.32	−2, –8, 38

vmPFC: ventromedial prefrontal cortex; BA: Brodmann’s area; OCD: obsessive-compulsive disorder; PFC: prefrontal cortex; ACC: anterior cingulate cortex.

Brain regions show significant connectivity to the seed region.

Thresholds were set at p < 0.001 (uncorrected), cluster threshold = 10 voxels.

a White matter tracts implicated.

**Figure 3.**
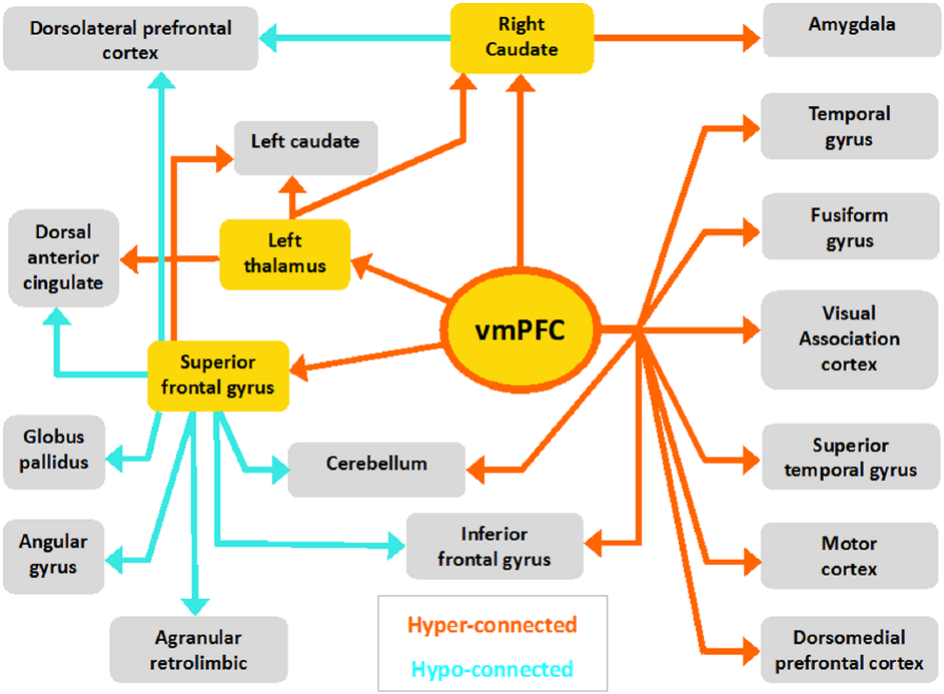
Schematic map of altered functional connectivity in OCD. Corresponding MNI coordinates and BA can be found in Tables 2 and 3.

Visual representation of the connectivity between brain regions comprising all seed-based analyses is summarised in Figure 3. All seed regions analysed are highlighted in yellow. Orange arrows indicate a significant increase in connectivity from these seeds in OCD patients compared to controls (OCD > Control). Turquoise arrows indicate decreased connectivity in OCD as compared to controls (OCD < Control). Corresponding z-values and coordinates are listed in Table 3.

### Medication

Minor group differences in connectivity were found when comparing unmedicated (N = 13) and medicated (N = 25) patients with OCD (all ps < 0.001 uncorrected). Unmedicated patients showed increased connectivity compared to medicated patients (unmedicated > medicated), from the vmPFC to the left insula (−44, 2, 0; cluster size = 54 voxels, peak z-score = 3.68), extending into the left primary sensory cortex (−34, −26, 20; cluster size = 33 voxels, peak z-score = 3.52). No significant increases in connectivity were found for the medicated > unmedicated contrast. Hyperconnectivity with the insula was also found in unmedicated patients when seeding from the caudate (38, 26, 2; cluster size = 34 voxels, peak z-score = 3.52) and the superior frontal gyrus (34, 28, 2; cluster size = 61 voxels, peak z-score = 3.78), suggesting a role for SSRIs in dampening hyperconnectivity with the insula. Medicated (N = 25) and unmedicated (N = 13) patients were matched on clinical characteristics.

## Discussion

### Widespread vmPFC hyperconnectivity

Our results confirm that the vmPFC is hyperconnected during rest in patients with OCD, displaying increased connectivity with its own surrounding region in addition to hyperconnectivity with several areas along the TCS loop: thalamus, caudate and frontal gyrus. OCD patients also exhibited increased functional connectivity from the vmPFC to temporal and occipital lobes, cerebellum and the motor cortex. These network alterations are therefore not limited to the TCS-circuitry, but reflect alterations in vmPFC connectivity in large-scale brain networks. No significant increases in vmPFC connectivity were observed in controls relative to patients, indicating one-sided widespread hyperconnectivity of the vmPFC in OCD.

In our sample, vmPFC auto-hyperconnectivity in OCD correlated with symptomatology related to neutralising scores. From studies with healthy participants, we know that the vmPFC deactivates during externally directed attention (Uddin et al., 2013), while the vmPFC of OCD patients fails to deactivate during tasks (Fitzgerald et al., 2005; Maltby et al., 2005, although see Milad et al. (2013)). These findings support the idea that the vmPFC is over-recruited by self-referential thinking and is therefore unavailable for effective valuation of external stimuli (Stern et al., 2013). In concordance with our previous findings (Apergis-Schoute et al., 2017), we show that in the same patients whose vmPFC fails to deactivate during task performance also exhibits intrinsic vmPFC hyperconnectivity at rest. The widespread hyperconnectivity of the vmPFC found in OCD patients at rest indicates an intrinsic network influence of vmPFC hyperconnectivity, likely undermining effective valuation, which is reflected in impairments in a variety of tasks (Haber and Heilbronner, 2013)

### Hyperconnectivity with the caudate and its relation to compulsivity

In OCD, the maintenance of habits has been shown to be associated with hyperactivity of the caudate nucleus (Gillan et al., 2015). Although we did not find widespread hyperconnectivity of the caudate itself, we showed that brain regions such as the vmPFC, superior frontal gyrus (SFG) and thalamus exhibited hyperconnectivity with the caudate in OCD patients. Although aberrant functional activity of the caudate is a very consistent finding in the literature on OCD (reviewed in Milad and Rauch (2012)), there have been inconsistencies in the directionality of findings, meaning that both hyper- and hypoactivation patterns have been observed (Abramovitch et al., 2015).

Our findings replicate hyperconnectivity of the vmPFC with the caudate (Chen et al., 2016), and we know from diffusion tensor imaging (DTI) that there is a strong structural connection between the vmPFC and the caudate, which is likely altered in OCD (de Wit et al., 2012). In our study, the strength of functional connectivity between the vmPFC and the caudate correlated with Y-BOCS scores, indicating a relation with OCD symptomatology.

Correlations of resting-state connectivity with OCD symptomatology have previously been observed in the vmPFC itself (Le Jeune et al., 2010), as well as in OFC connectivity (Meunier et al., 2012), and with OFC-caudate connectivity (Harrison, 2009). The self-reported urge to perform habits in OCD has been shown to be associated with hyperactivity of the caudate, (Gillan et al., 2015). As the vmPFC is structurally connected to the caudate, and the caudate has strong projections to and from the motor cortex (De Wit et al., 2012; Robinson et al., 2012), we speculate that within this habit/goal-directed circuitry, increased connectivity with the caudate may result in failures in linking actions to their consequences in OCD, promoting over-reliance on habits and having negative effects on other goal-directed behaviour needed in everyday life, reflected in impairments in various cognitive tests (Gillan and Robbins, 2014).

### Exploratory connectivity analyses of the OCD network

Building on the results from our main hypothesis, displaying widespread vmPFC hyperconnectivity in OCD, we investigated three additional seed regions in the TCS loop showing hyperconnectivity with the vmPFC. The connectivity schematic of these additional seed regions (caudate, thalamus and superior frontal cortex) provides a visual representation of vmPFC network connectivity in OCD at rest. As this analysis uses three supplementary TCS seed regions resulting from the initial hyperconnectivity with our vmPFC seed region, these results should be considered exploratory.

Our finding of vmPFC-caudate hyperconnectivity correlating with OCD symptomatology and the exploratory analysis showing hyperconnectivity with the caudate provides additional evidence that the caudate is a critical node in the TCS loop, and further substantiates the central role for the caudate in relation to compulsivity. It is important to note that our seed regions project to different areas of the caudate. fMRI does not lend itself to investigate differential neuronal projections, though it is known the caudate has distinct dorsal and ventral connectivity patterns (Harrison et al., 2009), and it is plausible that separate subparts of the caudate play unique roles in compulsive behaviour.

The orbito-frontal cortex is also widely mentioned as a critical region in OCD (Chamberlain et al., 2008; Graybiel and Rauch, 2000; Hou et al., 2014; Saxena et al., 1998); however, the vmPFC was the only frontal cortical area that displayed hyperconnectivity in our analysis and was not significantly hyperconnected to the OFC (Meunier et al., 2012). Other frontal regions, the SFG (as well as the inputs to the dlPFC and to some degree the dmPFC), all showed hypoconnectivity (Vaghi et al., 2016). While taking caution with making inferences of the impact of resting-state connectivity results on behaviour, widespread vmPFC hyperconnectivity together with impaired orbito- and lateral frontal cortical connectivity (Chamberlain et al., 2008; Vaghi et al., 2016) in OCD highlights dysfunction of regions associated with habit learning, inhibitory control, information processing, attention, self-awareness and mental imagery (Aron et al., 2003; Van Velzen et al., 2014).

Processes such as attention, self-awareness and mental imagery are related to the default mode network, in which the vmPFC is a central hub (Buckner et al., 2008). Confirming substantial alterations of the DMN in OCD (Beucke et al., 2014), we found hyperconnectivity of the vmPFC with the dmPFC and temporal cortex, hyperconnectivity of the caudate with the hippocampus and hyperconnectivity of the thalamus with the ACC.

Decreased connectivity was found between the SFG and both the parietal lobes and the dlPFC. We hypothesise that aberrations in the DMN, with a central role for the vmPFC, could drive intrusive thoughts and imagery, undermining external valuation and integration.

### Influences of medication on vmPFC connectivity

SSRI treatment has been shown to alter resting-state connectivity, highlighting the importance of medication profiles when interpreting results (Shin et al., 2014). Medication did not drive our main findings although patients on SSRIs showed attenuated hyperconnectivity of the vmPFC with the insula when compared to unmedicated patients. The salience network is important in assessing the relevance of internal and external stimuli in order to guide behaviour (Seeley et al., 2007). Our previous findings on threat reversal learning also showed task-related hyperconnectivity of the vmPFC with the insula as part of the salience network, indicating increased processing of threat in OCD. In this task, OCD patients showed generalisation during reversal alongside impairments in differential threat (CS+) versus safety (CS−) signalling in the insula (Apergis-Schoute et al., 2017). Further research should be conducted to investigate the effects of SSRIs on rs-FC, as well as its relation to symptom improvement in OCD.

## Conclusion

Taken together, this study confirms abnormal rs-FC in patients with OCD. The vmPFC was found to be widely hyperconnected, confirming its central role in the neuropathology of OCD. Specifically, the correlation of OCD symptomatology with vmPFC-caudate hyperconnectivity highlights its relation with obsessions and compulsivity.

Furthermore, the thalamus, superior frontal gyrus and vmPFC all show hyperconnectivity with the caudate, confirming maladaptive crosstalk of the caudate in the disorder. These results support the notion of a hyperconnected ventral medial prefrontal pathway and hypoconnected orbito- and lateral prefrontal cortex-striatal pathways in OCD.
